# Decline of Supportive Attitudes among Husbands toward Female Genital Mutilation and Its Association to Those Practices in Yemen

**DOI:** 10.1371/journal.pone.0083140

**Published:** 2013-12-18

**Authors:** Ghadah Abdulmajid Al-Khulaidi, Keiko Nakamura, Kaoruko Seino, Masashi Kizuki

**Affiliations:** 1 Department of International Health and Medicine, Division of Public Health, Graduate School of Tokyo Medical and Dental University, Tokyo, Japan; 2 Department of Health Promotion, Division of Public Health, Graduate School of Tokyo Medical and Dental University, Tokyo, Japan; Universität Bochum, Germany

## Abstract

**Objectives:**

To elucidate the attitudes of women and their husband’s towards female genital mutilation (FGM) and their associations with the continuation of FGM upon their daughters.

**Methods:**

Subjects were 10,345 (in 1997) and 11,252 (in 2003) ever married women aged 15 to 49 years from the Yemen Demographic Health Surveys. Performances of FGM on the most-recently-born daughters were investigated. Attitudes of women and their husbands were assessed by their opinions on the continuation of FGM. The association between the attitudes of women and their husbands and performance of FGM on the most-recently-born daughters were investigated after adjusting for age and education of the women.

**Findings:**

The percentage among the most-recently-born daughters who received FGM of women who had undergone FGM declined from 61.9% in 1997 to 56.5% in 2003 (p<0.001). The percentages of women who had undergone FGM and who supported the continuation of FGM and of husbands who also supported its continuation decreased from 78.2% and 60.1% in 1997 to 70.9% and 49.5% in 2003, respectively (both p<0.001). When the women or the husbands did not agree with FGM, it was less likely to be performed on their daughter than when the women or the husbands agreed in 1997 (odds ratio=0.11, 95% confidence interval 0.07-0.16 and odds ratio=0.07, 95% confidence interval 0.04-0.12, respectively) and in 2003 (odds ratio=0.12, 95% confidence interval 0.09-0.16 and odds ratio=0.11, 95% confidence interval 0.07-0.16, respectively).

**Conclusion:**

Non-supportive attitudes of women and their husbands towards the continuation of FGM have become common and were associated with their decision not to perform FGM upon their daughters.

## Introduction

Female genital mutilation (FGM) comprises all procedures involving partial or total removal of the external female genitalia or other intentional injuries to the female genital organs for non-medical reasons. FGM is also known as female circumcision and female genital cutting. About 140 million girls and women worldwide are estimated to be living with the consequences of FGM, and more than 2 million girls are suggested to be at risk of undergoing the procedures every year [1,2].

The World Health Organization (WHO) reported that women who have had FGM are significantly more likely to experience difficulties during childbirth, and their babies are more likely to die as a result of the practice [1,3,4]. Immediate health risks of FGM include severe pain, shock, excessive bleeding, difficulty in passing urine, infection, Human Immunodeficiency Virus (HIV), death, psychological consequences, unintended labial fusion, and repeated FGM. Long-term consequences include pain, infection, keloid, HIV, reproductive tract infections and sexually transmitted infections, birth complication, danger to the new-born, psychological consequences, quality of sexual life, infertility, later surgery, painful sexual intercourse, and urinary and menstrual problems [5]. Moreover, FGM is usually performed by non-professionals who do not even know the anatomy of the female genitalia [3,4].

Every major international health and rights consensus document of the last decade condemns FGM, and the Programme of Action of the International Conference on Population and Development (ICPD) calls on governments “to prohibit female genital mutilation wherever it exists and to give vigorous support to efforts among nongovernmental and community organizations and religious institutions to eliminate such practices” (paragraph 4.22) [1,5]. In 2008, the World Health Assembly adopted resolution WHA61.16 on the elimination of FGM, in which all member states agreed to work towards the abandonment of FGM, including ensuring that the procedure is not performed by health professionals [6]. 

The prevailing reasons for FGM are complex and numerous, and include the sociological (initiation of girls into womanhood), social integration and the maintenance of social cohesion; hygienic and aesthetic (belief that the female genitalia are dirty and unsightly); sexual (control or reduction of female sexuality); health (belief that it enhances fertility and child survival); religious (belief that it is a religious requirement); socio-economic factors (belief that FGM is a prerequisite for marriage, and where women are largely dependent on men, economic necessity can be a determinant for undergoing the procedure, and the practice can also be a major source of income for circumcisers); migration and displacement [7,8].

A woman’s attitude towards FGM—is very much related to her status as a woman within her community [9]. According to Maja and Litt, men are often left out of the picture when decisions about reproductive health issues are discussed, and male involvement in reproductive health should be mainstreamed in all major thrusts of the strategic framework, and men of all ages must be educated about responsible sexual behaviour [10].

FGM is performed in every social stratum, among both rich and poor, undereducated and highly educated, and both in cities and the countryside [11]. Countries may pass laws to eradicate FGM, but legal instruments by themselves cannot end the practice due to strong and deeply rooted traditions and beliefs in societies [12].

This study aims to highlight the attitudes of women of reproductive age (15-49 years old) and their husbands towards the practice of FGM, as well as their association with the performance of FGM upon their daughters. This is one of the first studies using Demographic Health Survey (DHS) data to show practice of FGM in Yemen from 1997 to 2003.

## Methods

### Ethics statement

Not applicable.

### Data source

The DHS programme was designed as a follow-up to the World Fertility Survey and the Contraceptive Prevalence Survey projects. The programme has been implemented in overlapping five-year phases. In Yemen, the survey was implemented by the Central Statistical Organisation/Ministry of Planning & International Cooperation in 1997 and by the Ministry of Public Health and Population and the Central Statistical Organisation/Ministry of Planning & International Cooperation, under the supervision of the Arab Family Health Survey in 2003. Permission to use Yemen’s DHS data from 1997 and 2003 for the analysis of health status of women and related conditions was provided by the Yemen Central Statistical Organisation/Ministry of Planning & International Cooperation.

### Sampling

The DHS was designed on the basis of the multi-stage sampling in order to provide estimates for general indicators for Yemen as a whole, for urban areas, and for rural areas. Islands were excluded from a sampling frame because of the small sizes of their populations and the difficulty in accessing them [13,14].

The data we extracted from the DHS database for the present analysis were 10,345 and 11,252 women of reproductive age (aged 15-49) who had ever married, in 1997 and 2003 respectively.

### Measures and procedure

We collected information about characteristics of women (age and education) and FGM (if they had ever heard about FGM). The questions related to FGM were asked only of women who had heard of FGM. For women who reported that they had ever heard about FGM, further questions were asked about women and their husband’s attitudes toward FGM, and if they had performed FGM on their most-recently-born daughters. The attitudes were assessed by asking respondents their opinions and their perceptions of their husband’s opinions on the continuation of FGM. The response alternatives for this query were “practice should be continued”, “practice should be stopped,” “don’t know”, and the additional alternative “attitude not clear” only for the husbands attitude.

The variables of age, education, having heard of FGM, having ever undergone FGM, the performance of FGM on the most-recently-born daughter, the respondent’s attitude, and husband’s attitude towards FGM were used for the analysis. In addition where FGM took place, who performed the FGM, and the age of the most-recently-born daughter when she received FGM were reported. 

### Statistical analysis

The percentage of performance of FGM on the most-recently-born daughters and attitudes of women and their husbands toward FGM were compared between 1997 and 2003 by using chi-square test.

Logistic regression analyses were applied to investigate the association of attitudes of women and their husbands towards FGM and performance of FGM on the most-recently-born daughters among women who had undergone FGM and women who hadn’t undergone FGM separately. The independent variables were age and education of women and women’s and husband’s attitudes towards the continuation of FGM. These analyses were done separately for 1997 and 2003.

To investigate independent association of the year of survey on performance of FGM on the most-recently-born daughters, another logistic regression model was applied after pooling data from two survey years. 

Sample weight was applied to correct disproportional sampling probabilities and to adjust the collected data to represent the population from which the sample was drawn. Incomplete data on FGM related and other variables were excluded from the analyses. The level of statistical significance for all analysis was set at α=0.05. All statistical analyses were performed with SPSS version 18.

## Results

Table 1 shows the distribution of demographic variables and FGM related variables in 1997 and 2003. The percentage of illiterate women was lower in 2003 than in 1997 (p<0.001). The percentage of women who had heard of FGM was higher in 2003 than in 1997 (p<0.001). The percentage of most-recently-born daughters who received FGM was 29.3% in 1997 and 22.4% in 2003, and significantly lower in 2003 than in 1997 (p<0.001). More women and more of their husbands thought that FGM practice should be stopped in 2003 than in 1997 (both p<0.001). The percentage of couples that both agreed that the FGM practice should be continued was reduced from 27.5% in 1997 to 19.6% in 2003 (p<0.001). 

**Table 1:** Age and education of women and attitude and practice related with female genital mutilation (FGM) in Yemen, 1997 and 2003.

			**1997**			**2003**			**p**
			**n** *	**%**		**n** *	**%**		
**Among all women aged 15-49**									
**Total**			**10345**	**100.0**		**11252**	**100.0**		
**Age**									<0.001
	15-19		1101	10.6		865	7.7		
	20-24		1986	19.2		2206	19.6		
	25-29		1927	18.6		2198	19.5		
	30-34		1669	16.1		1681	14.9		
	35-39		1757	17.0		1813	16.1		
	40-44		1083	10.5		1389	12.3		
	45-49		822	7.9		1099	9.8		
**Education**									<0.001
	Illiterate		8429	81.5		8688	77.2		
	Literate		1916	18.5		2564	22.8		
**Women have heard about FGM**									<0.001
	Yes		5226	50.5		6301	56.0		
	No		5119	49.5		4951	44.0		
**Among women aged 15-49 who ever heard of FGM**									
**Total**			**5226**	**100.0**		**6302**	**100.0**		
**Women have undergone FGM**									<0.001
	Yes		2335	44.7		2420	38.4		
	No		2891	55.3		3882	61.6		
**Most-recently-born daughter undergone FGM**									<0.001
	Yes		1530	29.3		1413	22.4		
	No		2449	46.9		3452	54.8		
	No daughters		1247	23.9		1436	22.8		
**Women's attitude towards FGM**									<0.001
	Practice should be continued		2143	41.0		2034	32.3		
	Practice should be stopped		2502	47.9		3426	54.4		
	Do not know		581	11.1		842	13.4		
**Husband's attitude towards FGM**									<0.001
	Practice should be continued		1557	29.8		1332	21.1		
	Practice should be stopped		1030	19.7		1375	21.8		
	Attitude not clear		113	2.2		167	2.6		
	Do not know		2197	42.0		3034	48.2		
	No husband		330	6.3		394	6.2		
**Woman's and husband's attitude towards FGM**									<0.001
	Both agree		1437	27.5		1233	19.6		
	Husbands disagree and women agree		77	1.5		115	1.8		
	Husbands agree and women disagree		80	1.5		67	1.1		
	Both disagree		912	17.4		1208	19.2		
	Do not know		2442	46.7		3344	53.1		
	No husband		278	5.3		335	5.3		
**Among most-recently-born daughter who underwent FGM**									
**Total**			**1530**	**100.0**		**1413**	**100.0**		
**Age when FGM was performed^*#*^**									<0.001
	≥ birth, < 1 month		1091	92.8		1091	81.1		
	≥ 1 month, < 1 year		76	6.5		250	18.6		
	≥ 1 year		8	0.7		4	0.3		
	Other		1	0.1		0	0.0		
**Location where FGM took place^*#*^**									<0.001
	At home		1140	96.9		1277	94.8		
	Health facility		37	3.1		54	4.0		
	Other		0	0.0		17	1.3		
**Person performing FGM^*#*^**									<0.001
	Health provider		112	9.6		155	11.5		
	Traditional birth attendant		841	71.4		805	59.8		
	Traditional healer		177	15.0		203	15.1		
	Barber		42	3.6		142	10.6		
	Other		5	0.4		42	3.1		

p-values were calculated with chi-square tests.

^*^ After sampling weights were applied, numbers were rounded.

^#^ Due to missing cases, the sum number of cases by categories are not equal to the total number of cases.

The DHS data showed that FGM was performed on the most-recently-born daughter mainly within the first month after birth (92.8% in 1997 and 81.1% in 2003), and at home (96.9% in 1997 and 94.8% in 2003). In 71.4% of the cases, FGM was performed by a traditional birth attendance (daya or Kharshofa) in 1997; and this percentage dropped to 59.8% in 2003.

Table 2 shows FGM practice on most-recently-born daughters and the attitudes of women and their husbands for women who had undergone FGM and for women who had not. Women who had undergone FGM were more likely to perform FGM on their daughters (61.9% in 1997 and 56.5% in 2003, p<0.001) and were more likely to be supportive of FGM continuation (78.2% and 70.9%, p<0.001) than women who had not. Husbands of women who had undergone FGM were supportive of FGM continuation (60.1% and 49.5%, p<0.001). Both the women and their husbands were more supportive regarding performance of FGM when the women had not undergone FGM (57.5% and 47.4%, p<0.001). 

**Table 2:** FGM practice on most-recently-born daughter and attitudes towards FGM of women and their husbands for women who had undergone FGM and women who had not.

			**1997**				**p**		**2003**				**p**
			**Women had undergone FGM**						**Women had undergone FGM**				
			**Yes**		**No**				**Yes**		**No**		
			**n**	**%**	**n**	**%**			**n**	**%**	**n**	**%**	
**Most-recently-born daughter underwent FGM**							<0.001						<0.001
	Yes		1445	61.9	85	2.9			1366	56.5	47	1.2	
	No		321	13.7	2128	73.6			512	21.2	2940	75.7	
	No daughters		569	24.4	678	23.5			541	22.4	895	23.1	
**Women's attitude towards FGM**							<0.001						<0.001
	Practice should be continued		1827	78.2	316	10.9			1715	70.9	319	8.2	
	Practice should be stopped		387	16.6	2115	73.2			547	22.6	2879	74.2	
	Do not know		121	5.2	460	15.9			159	6.6	683	17.6	
**Husbands' attitude towards FGM**							<0.001						<0.001
	Practice should be continued		1403	60.1	154	5.3			1199	49.5	133	3.4	
	Practice should be stopped		212	9.1	818	28.3			341	14.1	1034	26.6	
	Attitude not clear		54	2.3	59	2.0			67	2.8	100	2.6	
	Do not know		530	22.7	1667	57.6			657	27.1	2378	61.3	
	No husband		136	5.8	194	6.7			157	6.5	237	6.1	
**Woman's and husband's attitude towards FGM**							<0.001						<0.001
	Both agree		1342	57.5	96	3.3			1147	47.4	86	2.2	
	Husbands disagree and women agree		40	1.7	37	1.3			79	3.3	35	0.9	
	Husbands agree and women disagree		35	1.5	45	1.6			36	1.5	31	0.8	
	Both disagree		163	7.0	748	25.9			252	10.4	957	24.6	
	Do not know		630	27.0	1812	62.6			761	31.4	2583	66.5	
	No husband		125	5.3	154	5.3			146	6.0	189	4.9	

Table 3 shows the adjusted association of women and their husband’s attitude towards FGM with FGM being performed on the most-recently-born daughter among women who undergone FGM in 1997 and 2003, separately. When women did not agree with FGM, daughters were less likely to receive FGM in 1997 (odds ratio 0.11, 95% confidence interval 0.07-0.16) and in 2003 (odds ratio 0.12, 95% confidence interval 0.09-0.16). When husbands did not agree with FGM, daughters were less likely to receive FGM in 1997 (odds ratio 0.07, 95% confidence interval 0.04-0.12) and in 2003 (odds ratio 0.11, 95% confidence interval 0.06-0.17). 

**Table 3:** Associations of age and education of women, woman's and husband's attitude towards female genital mutilation (FGM), and FGM performance on most-recently-born daughter among women who undergone FGM in Yemen in 1997 and 2003.

		**1997 (n=2335)**						**2003 (n=2420)**				
		**FGM on most-recently-born daughter**	**Adjusted OR**	**95%CI**		**p**		**FGM on most-recently-born daughter**	**Adjusted OR**	**95%CI**		**p**
		**%**		**Lower limit**	**Upper limit**			**%**		**Lower limit**	**Upper limit**	
**Age**												
	15-19	81.1	1.21	0.45	3.25	0.701		78.2	1.02	0.33	3.17	0.969
	20-24	75.8	0.53	0.27	1.04	0.066		71.0	0.81	0.47	1.37	0.423
	25-29	82.6	1.04	0.54	2.02	0.897		72.4	0.88	0.54	1.44	0.621
	30-34	81.5	1.09	0.58	2.05	0.792		74.1	0.88	0.54	1.44	0.619
	35-39	83.4	0.81	0.43	1.53	0.515		71.5	0.87	0.55	1.40	0.572
	40-44	83.6	1.06	0.54	2.08	0.863		72.1	0.80	0.49	1.30	0.367
	45-49	83.3	reference					75.0	reference			
**Education**												
	Illiterate	84.5	reference					73.9	reference			
	Literate	68.2	0.40	0.26	0.61	<0.001		67.9	0.99	0.71	1.37	0.936
**Woman's attitude toward FGM**												
	Practice should be continued	94.1	reference					89.0	reference			
	Practice should be stopped	35.2	0.11	0.07	0.16	<0.001		26.8	0.12	0.09	0.16	<0.001
	Do not know	59.2	0.20	0.12	0.34	<0.001		57.8	0.33	0.21	0.52	<0.001
**Husband's attitude toward FGM**												
	Practice should be continued	96.7	reference					92.6	reference			
	Practice should be stopped	30.2	0.07	0.04	0.12	<0.001		26.9	0.11	0.07	0.16	<0.001
	Attitude not clear	76.7	0.36	0.14	0.90	0.029		65.5	0.29	0.15	0.55	<0.001
	Do not know	61.1	0.12	0.08	0.18	<0.001		58.5	0.25	0.18	0.36	<0.001
	No husband	82.8	0.27	0.13	0.54	<0.001		69.5	0.38	0.22	0.66	<0.001

Adjusted odds ratios (ORs) were estimated using multivariable logistic regression analysis. The model included all variables in this table as independent variables.

Table 4 shows the adjusted association of women and their husband’s attitude toward FGM with FGM being performed on the most-recently-born daughter among women who hadn't undergone FGM in 1997 and 2003, separately. When women did not agree with FGM, daughters were less likely to receive FGM in 1997 (odds ratio 0.13, 95% confidence interval 0.07-0.23) and in 2003 (odds ratio 0.14, 95% confidence interval 0.06-0.33). When husbands did not agree with FGM, daughters were less likely to receive FGM in 1997 (odds ratio 0.14, 95% confidence interval 0.06-0.31) and in 2003 (odds ratio 0.12, 95% confidence interval 0.04-0.38).

**Table 4:** Associations of age and education of women, woman's and husband's attitude towards female genital mutilation (FGM), and FGM performance on most-recently-born daughter among women who hadn't undergone FGM in Yemen in 1997 and 2003.

		**1997 (n= 2892)**						**2003 (n=3882)**				
		**FGM on most-recently-born daughter**	**Adjusted OR**	**95%CI**		**p**		**FGM on most-recently-born daughter**	**Adjusted OR**	**95%CI**		**p**
		**%**		**Lower limit**	**Upper limit**			**%**		**Lower limit**	**Upper limit**	
**Age**												
	15-19	4.9	2.72	0.64	11.55	0.176		2.6	2.02	0.30	13.77	0.474
	20-24	2.1	0.47	0.16	1.44	0.187		1.7	1.34	0.40	4.50	0.638
	25-29	3.8	1.00	0.40	2.48	0.999		1.4	0.89	0.27	2.88	0.842
	30-34	2.9	0.69	0.26	1.81	0.450		1.8	0.86	0.27	2.70	0.797
	35-39	6.2	1.32	0.57	3.05	0.518		1.6	0.68	0.21	2.19	0.521
	40-44	2.2	0.50	0.16	1.62	0.251		1.3	0.41	0.11	1.51	0.181
	45-49	5.2	reference					1.6	reference			
**Education**												
	Illiterate	4.4	reference					1.9	reference			
	Literate	2.0	0.59	0.28	1.27	0.177		0.7	0.32	0.12	0.83	0.019
**Woman's attitude toward FGM**												
	Practice should be continued	22.8	reference					10.8	reference			
	Practice should be stopped	1.5	0.13	0.07	0.23	<0.001		0.7	0.14	0.06	0.33	<0.001
	Do not know	2.2	0.20	0.08	0.46	<0.001		1.0	0.16	0.06	0.47	0.001
**Husband's attitude toward FGM**												
	Practice should be continued	33.5	reference					18.9	reference			
	Practice should be stopped	2.0	0.14	0.06	0.31	<0.001		0.7	0.12	0.04	0.38	<0.001
	Attitude not clear	13.4	0.51	0.19	1.37	0.180		4.8	0.66	0.19	2.25	0.502
	Do not know	1.0	0.06	0.03	0.12	<0.001		0.9	0.11	0.05	0.26	<0.001
	No husband	7.9	0.43	0.19	0.97	0.043		0.6	0.08	0.01	0.61	0.015

Adjusted odds ratios (ORs) were estimated using multivariable logistic regression analysis. The model included all variables in this table as independent variables.

Table 5 shows the adjusted differences in the performance of FGM on the most-recently-born daughter between survey years after combining 1997 and 2003 data of women’s experience in having undergone FGM. Daughters were less likely to receive FGM in 2003 than in 1997 among women who had undergone FGM (odds ratio 0.72, 95% confidence interval 0.58–0.88) after adjustment for age and education of women and attitudes of women and their husbands. Moreover, the odds ratio for performance of FGM on the most-recently-born daughter among women who hadn't undergone FGM showed further decline of FGM on daughters from 1997 to 2003 (odds ratio 0.53, 95% confidence interval 0.36–0.80). 

**Table 5:** Reduction of performance of female genital mutilation (FGM) on most-recently-born daughter in Yemen from 1997 to 2003* adjusted for agreement and education of women, by women’s experience in having undergone FGM.

		**Adjusted OR**	**95%CI**		**p**
			**Lower limit**	**Upper limit**	
**FGM on most-recently-born daughter among women who had undergone FGM** (**n=4755**)					
**Year**					
	1997	reference			
	2003	0.72	0.58	0.88	0.001
**Age**					
	15-19	1.40	0.69	2.83	0.353
	20-24	0.68	0.46	1.02	0.060
	25-29	0.92	0.63	1.33	0.642
	30-34	0.94	0.65	1.36	0.734
	35-39	0.89	0.62	1.28	0.534
	40-44	0.91	0.62	1.34	0.624
	45-49	reference			
**Education**					
	Illiterate	reference			
	Literate	0.69	0.54	0.89	0.004
**Woman's and husband's attitude towards FGM**					
	Both agree	reference			
	Husbands disagree and women agree	0.03	0.02	0.05	<0.001
	Husbands agree and women disagree	0.13	0.07	0.23	<0.001
	Both disagree	0.02	0.01	0.02	<0.001
	Do not know	0.07	0.06	0.10	<0.001
**FGM on most-recently-born daughter among women who hadn't undergo FGM (n=6774)**					
**Year**					
	1997	reference			
	2003	0.53	0.36	0.80	0.002
**Age**					
	15-19	2.08	0.68	6.39	0.203
	20-24	0.71	0.31	1.61	0.409
	25-29	0.83	0.39	1.75	0.622
	30-34	0.74	0.35	1.57	0.431
	35-39	0.95	0.47	1.93	0.892
	40-44	0.48	0.20	1.16	0.103
	45-49	reference			
**Education**					
	Illiterate	reference			
	Literate	0.48	0.27	0.87	0.015
**Woman's and husband's attitude towards FGM**					
	Both agree	reference			
	Husbands disagree and women agree	0.19	0.07	0.53	0.002
	Husbands agree and women disagree	0.26	0.11	0.62	0.002
	Both disagree	0.02	0.01	0.04	<0.001
	Do not know	0.03	0.02	0.04	<0.001

^*^ Reduction was shown as odds ratios (ORs) for year comparing 2003 with 1997.

## Discussion

The results showed that frequency of FGM on daughters has declined from 1997 to 2003. During this period, the awareness of FGM practice has increased among women. Women who undergone FGM were more likely to have it performed on their daughters. Women’s and husbands’ attitudes not supportive to the continuation of FGM was significantly associated with not having performed FGM on their daughters regardless of women’s age and education.

There was a decline in the FGM supportive attitudes and practice in the population from 1997 to 2003. The reduction in practice over the years was partly explained by the changes of women’s and their husbands’ attitudes towards FGM, and improvement in education among women from 1997 to 2003. The changes in attitudes of women and their husbands could be due to the ministerial decree prohibiting health providers from performing FGM in Yemen. This ministerial decree was a result of the Clinical-Based Investigation of Female Genital Mutilation in Selected Areas of Yemen in 1999 by different government entities. However, health officials mentioned that the decree’s effectiveness couldn’t be monitored in all medical facilities [3]. Recent exposure by the population to the information from health providers, media, and health projects that help in raising awareness on health issues, including FGM could also have contributed to the change of attitudes of people in Yemen towards FGM. Literate women were less likely to perform FGM on their daughters than illiterate women. Access to education can help end FGM because there is a significant relationship between education and FGM. The attitude of the illiterate women was more supportive towards FGM than that of literate women. It was reported that the more educated the mothers are, the less likely they are to have their daughters circumcised [15]. Education in the case of FGM is seen as a source of empowerment for women because it can facilitate their abilities to gather and assimilate information [16]; this point agrees with other studies that the practice of FGM and support for its discontinuation by women changes with education and awareness.

This study showed that the attitudes of husbands are also important. A daughter is more likely to receive FGM when the attitude towards FGM of her father is positive. The roles of women’s attitudes to FGM in decision making are complex and differ based on the society. It is possible in some societies that women who think that FGM should be stopped should ensure that their own daughters undergo the procedure against their wish due to pressure from family and society. Yemen is a traditional society in which prevailing cultural attitudes bestow low status upon women in the family as well as in the community, and men are dominant [17–19]. Insufficient attention had been paid to men’s attitudes.

Men’s positive attitude towards FGM has declined. In 1998, WHO emphasized that males should be empowered through the provision of information and services targeting boys, youth, and adults within the home, community, and work setting [10,17,20,21]. In the past, men’s involvement has sometimes been opposed by women’s health advocates, who understandably fear that adding these services will damage the quality of women’s services and create additional competition for already scarce resources [11]. Since men were excluded most of the time from women’s reproductive health education and programmes, this could explain the lack of resources related to men attitude towards women’s reproductive health. Now, programmes on health education and women’s reproductive health include both the women and men.

Most FGM was performed at home by a traditional birth attendant and not a trained health provider. The practice depends on the community or individual family [7] and girls are not aware that they have been circumcised until they get married or have been examined. The practice at homes by a traditional birth attendant involves the use of unsterilized tools and instruments and the location could endanger the child’s life because it could be far from a health facility [1].

FGM is performed in early age of a girl’s life. The analysis of the DHS revealed that most of the practice of FGM in Yemen was performed within the first month after birth. This could be due to tradition and it could be to avoid pain. The daughters might not remember the immediate consequences or even did not know if she had been circumcised, but long-term risks will be faced by the girl when she grows up and get married. According to WHO, FGM involves removing and damaging healthy and normal female genital tissue, and interferes with the natural functions of girls’ and women’s bodies [1]. Women’s health is affected by their capacity to conceive, and this includes the risk of disorders associated with reproductive organs [22]. It was reported that pregnant women who had undergone FGM had risks at obstructed labour, which result in uterine rupture, with severe, often fatal bleeding, or postpartum vesical or rectal fistula [23]. 

There is a complex interplay between women and husbands attitudes [24]. Our analyses tease out the association of husbands’ attitudes with performance of FGM on daughters while controlling for wives’ attitudes.

This study measures the attitudes of husbands as perceived by their wives. There is a concern that the norms of women reflect social norms rather than the actual attitude of their husbands. However, in this study the likelihood of this was low because the women were given the options to answer “don’t know” or “not clear”. Therefore, the question on their husband’s attitudes did indeed measure it. 

Yemen is one of the developing countries selected as a pilot for the Millennium Development Goal (MDG) project. Yet, the country had concerns about achieving the MDG5 (improving maternal health) because of widely practiced FGM which has consequences of infection, pain, and trauma leading to negative health impacts. The health complications lead by FGM are associated with pregnancy, childbirth and the postpartum, which makes childbirth painful and dangerous as it prolongs labour, obstructs the birth canal and often causes perianal tears [25,26]. 

As a follow-up to the International Conference on Population and Development (ICPD), which states that governments are urged to prohibit female genital mutilation and to prevent infanticide [27], Yemen’s Ministry of Public Health and Population (MoPHP) drafted a law to prohibit female genital mutilation by anyone, including health providers in 2008. However, the law was not passed by parliament and challenged by questions raised by religious leaders in the Ministry of Endowment about the actual health consequences of FGM. Meantime in 2012, the MoPHP again drafted a law to prohibit female genital mutilation; in addition to educational and awareness programs on FGM by Yemeni government and partners for targeted population that started from 1999. 

ICPD also addressed sexual and reproductive health issues under chapter VII (Reproductive Rights and Reproductive Health) [27]. Studies and reviews confirm the need for sexual and reproductive health awareness and education for both men and women [28–30]. Therefore, there is a need for sexual and reproductive health education inducting the health risks from FGM at the community, school, and university levels. Men and boys should be included in measures addressing women’s health because they play a very important role in decision making. 

In conclusion, non-supportive attitudes by women and their husbands towards the practice of FGM influenced their decisions to abstain from having FGM performed upon their daughters. Significant factors in the decline in performance of FGM were changes in women’s and their husband’s attitudes towards FGM, and improved education of women.

## References

[1] World Health Organisation (2013). Fact sheet N.241. Available: http://www.who.int/mediacentre/factsheets/fs241/en/. Accessed: 09 August 2013

[2] United States Agency for International Development (2008) Numbers of Women Circumcised in Africa: The production of a Total. Calverton: Macro International Inc. 23 p.

[3] Fgmnetwork (2007). YEMEN: 24 percent of Yemeni women experience genital mutilation. The Female Genital Cutting Education and Networking Project. Available: http://www.fgmnetwork.org/gonews.php?subaction=showfull&id=1171338119&archive=&start_from=&ucat=1. Accessed 09 August 2013

[4] Cook R, Dickens B, Fathalla M (2010) Reproductive health and human rights, integrating medicine, ethics, and law. New York: Oxford University press Inc. pp. 262-266.

[5] World Health Organisation (2008) Eliminating Female Genital Mutilation: An interagency statement. OHCHR, UNAIDS, UNDP, UNECA, UNESCO, UNFPA, UNHCR, UNICEF, UNIFEM. Geneva: WHO press. 47 p.

[6] World Health Organisation (2010) Global strategy to stop health-care providers from perfuming female genital mutilation. UNAIDS, UNDP, UNFPA, UNICEF, UNHCR, UNIFEM, WHO, FIGO, ICN, IOM, WCPT, WMA, MWIA. Geneva: WHO Press. 17 p.

[7] FORWARD (2007). Female Genital Mutilation (FGM). Available: http://www.forwarduk.org.uk/key-issues/fgm/. Accessed 09 August 2013

[8] United Nations Population Funds (2007) A Holistic Approach to the Abandonment of Female Genital Mutilation/Cutting. New York: United Nation Population Fund. 20 p.

[9] Moore K, Randolph K, Toubia N, Kirberger E. (1997) The Synergistic Relationship between Health and Human Rights: A Case Study Using Female Genital Mutilation. Harvard School of Public Health Health and Human Rights 2(2): 137-146. 10381834

[10] Maja TMM, Litt D (2006). Men’s Involvement in Promoting Reproductive health. Women and Health Learning Package. Available: http://www.the-networktufh.org/sites/default/files/attachments/basic_pages/WHLP%20Men. Accessed 09 August 2013

[11] Avén J, Jacobson CA (2011). Nursing Students’ Knowledge of and Attitude towards Female Genital Mutilation: a quantitative study in Ghana. The Red Cross University College. Available: http://rkh.diva-portal.org/smash/record.jsf?pid=diva2:458813. Accessed 09 August 2013

[12] Ali AS (2010) The Role of Policymakers in Ending Female Genital Mutilation: An African Perspective. Population Reference Bureau. PRB: 4 p.

[13] Yemen Central Statistical Organisation (1997). Yemen Demographic Health Survey report. 292 p.

[14] Yemen Central Statistical Organisation and Ministry of Public Health and Population (2003). Yemen Demographic Health Survey Report. 264 p.

[15] Pashaei T, Rahimi A, Ardalan A, Felah A, Majlessi F (2012) Related factors of female genital mutilation (FGM) in Ravansar (Iran). Pashaei. J Women’s Health Care 1: 108.

[16] United Nations Children’s Fund (2005) Female Genital Mutilation/Cutting: A statistical exploration. New York: The United Nations Children’s Fund. 58 pp.

[17] Al-Shawafi N (1999) Clinical-Based Investigation of Female Genital Mutilation in Selected Areas in Yemen. Yemen: Ministry of Public Health and Population. 13 p.

[18] World Health Organisation (2006) Female genital mutilation—new knowledge spurs optimism. Progress newsletter NO. 72. Geneva: World Health Organisation.

[19] United Nation: Women watch (2010)Country Assessment on Violence against Women: case of; YEMEN. Available: http://www.un.org/womenwatch/ianwge/taskforces/vaw/Country_Assessment_on_Violence_against_Women_August_2_2010.pdf. Accessed 09 August 2013

[20] Rahman A, Toubia N (2000) Female Genital Mutilation: A Guide to Law and Policies Worldwide. United Kingdom and New York. Zed Books Ltd. pp. 5-7.

[21] Wegner MN, Landry E, Wilkinson D, Tzanis J (1998) Men as Partners in Reproductive Health: From Issues to Action; Special Report. Guttmacher Institute. International Family Planning Perspectives 24(1): 38-42. Available: http://www.guttmacher.org/pubs/journals/2403898.html. Accessed: 09 August 2013

[22] Payne S (2008) The Health of Men and Women. UK: Polity Press pp. 22-27.

[23] Lhbsu L (2009-2010). Female genital mutilation. United Kingdom: Midwives magazine. Available: http://www.rcm.org.uk/midwives/in-depth-papers/female-genital-mutilation/. Accessed 09 August 2013

[24] United Nations Children’s Fund (2013) Female Genital Mutilation/Cutting: A statistical overview and exploration of the dynamics of change. New York: United Nations Children’s Fund. 186 p.

[25] United Nation Population Fund (2009) Global Consultation on Female Genital Mutilation/Cutting. Technical Report. New York: United Nation Population Fund p. 112.

[26] United Nation Millennium Project (2002-2006) Pilot country inputs. Available: http://www.unmillenniumproject.org/reports/preface5.htm. Accessed 09 August 2013

[27] UN, United Nations Department of Public Information (1995). International Conference on Population and Development (ICPD): Summary of the Programme of Action. Available: http://www.un.org/popin/icpd/conference/offeng/poa.html. Accessed 09 August 2013

[28] Mohammadi G, Amiraliakbari S, Ramezankhani A, Majd HA (2011) Poor reproductive health among a group of socially damaged Middle Eastern women: a cross-sectional study. Int J Womens Health. 3: 399–403. PubMed: 22140328. 22140328 10.2147/IJWH.S26623 PMC3225470

[29] Vuttanont U, Greenhalgh T, Griffin M, Boynton P (2006) “Smart boys” and “sweet girls”—sex education needs in Thai teenagers- a mixed-method study. Lancet 368(9552): 2068-2080. doi:10.1016/S0140-6736(06)69836-X. PubMed: 17161729. 17161729 10.1016/S0140-6736(06)69836-X

[30] DeJong J, Jawad R, Mortagy I, Shepard B (2005) The sexual and reproductive health of young people in the Arab countries and Iran. Reprod Health Matters 13(25): 49-59. doi:10.1016/S0968-8080(05)25181-9. PubMed: 16035597. 16035597 10.1016/s0968-8080(05)25181-9

